# MetRec: A dataset for meter classification of arabic poetry

**DOI:** 10.1016/j.dib.2020.106497

**Published:** 2020-11-04

**Authors:** Maged S. Al-shaibani, Zaid Alyafeai, Irfan Ahmad

**Affiliations:** a Information and Computer Science Department, KFUPM, Dhahran 31261, Saudi Arabia

**Keywords:** Arabic, meter, poetry, classification, prosody

## Abstract

In this data article, we report a dataset related to the research titled “*Meter Classification of Arabic Poems Using Deep Bidirectional Recurrent Neural Networks”*[2]. The dataset was collected from a large repository of Arabic poems, *Aldiwan* website [1]. The data collection was done using a Python script that scrapes the website to find the poems and their associated meters. The dataset contains the verses and their corresponding meter classes. Meter classes are represented as numbers from 0 to 13. The dataset can be highly useful for further research in order to improve the field of Arabic poems’ meter classification.

## Specifications Table

**Table:** 

Subject	Artificial Intelligence
Specific subject area	Natural Language Processing
Type of data	Text
How data were acquired	The data was collected by scraping *Aldiwan* website [1]. The website is a large repository of Arabic poetry.
Data format	Raw text containing poems’ verses along their associated classes numbered as integers in the range of 0 to 13 representing the 14 poem meters.
Parameters for data collection	*Aldiwan* website [1] was scrapped to collect poems along with their meters. 14 m were collected.
Description of data collection	Python code was created to web-scrap the public webpages of the website. We developed a python script to collect these poems. Poems meters were also collected which were converted to integers between 0 and 13. Each class indicates a specific meter.
Data source location	The source of the data are the public web pages of Aldiwan website [1].
Data accessibility	Repository name: Arabic-Poetry Direct URL to data: https://raw.githubusercontent.com/zaidalyafeai/Arabic-Poetry/master/final_baits.zip
Related research article	Al-shaibani, M.S., Alyafeai, Z. and Ahmad, I., 2020. Meter Classification of Arabic Poems Using Deep Bidirectional Recurrent Neural Networks. Pattern Recognition Letters. DOI: 10.1016/j.patrec.2020.05.028

## Value of the Data

• Arabic Poetry is an important part of Arab heritage. The process of identifying Arabic poem meters is not straightforward. This dataset can be used for the purpose of automating this process.
• The dataset can be considered as a benchmark for Arabic poetry classification.
• Researchers can use the dataset to investigate further directions in the field. One possible research interest where this data can be useful is meter-based poem generation.
• To the best of our knowledge, there exists no public dataset for Arabic poems with their associated meters.

## 1 Data Description

The data is divided into two sets: training and testing. The training set is stored in the file named ‘train.txt’. It includes more than 47,124 rows. The file contains the rows formated as follows: Each row contains the verse and its meter id separated by a space. The row starts by the meter then the verse. The verse consists of two parts separated by a special character, ‘#’. Meters are encoded as class numbers from 0 to 13. These numbers refer to the order of these meters in ‘lables.txt’ file. Table 1 shows the meter labels and their corresponding names. The testing set is in the file named ‘test.txt’. It includes more than 8316 verses with their corresponding meters. The test set was formated in the similar way to the training set. The total number of rows in the full dataset is 55,440. In Fig. 1, we show the distribution of meters in the training set while in Fig. 2, we show the distribution in the test set.

**Table 1:** Meter label and its associated Name.

Meter Label	Meter name
0	*Saree*
1	*Kamel*
2	*Mutakareb*
3	*Mutadarak*
4	*Munsareh*
5	*Madeed*
6	*Mujtath*
7	*Ramal*
8	*Baseet*
9	*Khafeef*
10	*Taweel*
11	*Wafer*
12	*Hazaj*
13	*Rajaz*

**Fig.. 1:**
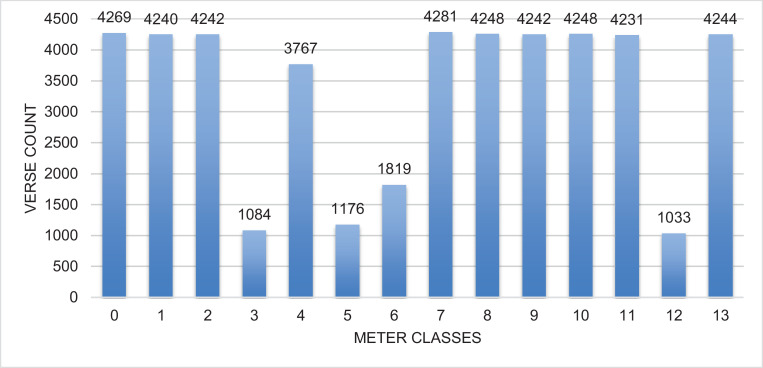
Meters Classes Distribution in the training set.

**Fig.. 2:**
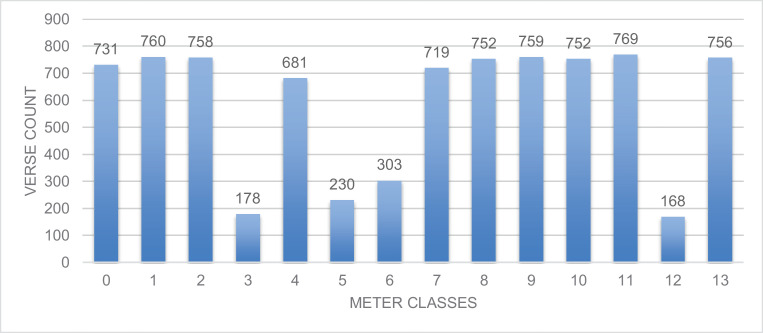
Meters Classes Distribution in the test set.

## 2 Experimental Design, Materials and Methods

The data is collected from *Aldiwan* website [1]. The website has a large collection of Arabic poems. The number of Arabic poem meters is sixteen in total. However, not all meters are commonly used in Arabic poetry. Some of the meters are highly used while others are less common. Additionally, there are two meters that are extremely rare. Some Arabic scholars consider them non-existent [3]. We did not include them in the dataset. The distribution for the meters in the training dataset is illustrated in Fig. 1. Three meters have less than 1200 verses while most of the meters have more than 4000 verses. Fig. 2 shows the distribution on the test set. The meter class ‘12′ corresponding to *Hazaj* meter has the least number of poem verses—1033 verses in the training set and 168 verses in the test set. Whereas, meter class ‘7′ corresponding to *Ramal* meter has the most number of poem verses—4281 verses—in the train set and the meter class ‘11′ corresponding to *Wafer* meter has the most number of poem verses—4281 verses—in the test set.

The website from where we collected the dataset classifies the poems according to their meters. All poems titles and links belonging to the same meter are accessible through a stand-alone web page. We scraped the website by accessing each meter page first to get its name and poems links. Each poem is then accessed to scrape its verses. The programming language used for this task is Python with *requests* library.

## Declaration of Competing Interest

The authors declare that they have no known competing financial interests of personal relationship that could have appeared to influence the work reported in this paper.

## Appendix A. Supplementary data

The dataset is accessible through this link: https://raw.githubusercontent.com/zaidalyafeai/Arabic-Poetry/master/final_baits.zip. Other materials are also accessible in this GitHub repository: https://raw.github.com/zaidalyafeai/Arabic-Poetry.
